# Active Propelled Micro Robots in Drug Delivery for Urologic Diseases

**DOI:** 10.3390/mi17010024

**Published:** 2025-12-25

**Authors:** Chunlian Zhong, Menghuan Tang, Zhaoqing Cong

**Affiliations:** 1 Department of Urology, Stony Brook University, Stony Brook, NY 11794, USA; chunlian.zhong@stonybrookmedicine.edu; 2 Stony Brook Cancer Center, Renaissance School of Medicine, Stony Brook University, Stony Brook, NY 11794, USA; 3 Department of Biochemistry & Molecular Medicine, University of California Davis, Sacramento, CA 95817, USA; tmhtang@ucdavis.edu

**Keywords:** active propulsion, micro robots, magnetic field, drug delivery, biohybrid, urologic disease, bladder cancer, kidney cancer

## Abstract

Active propelled micro robots (MRs) represent a transformative shift in biomedical engineering, engineered to navigate physiological environments by converting chemical, acoustic, or magnetic energy into mechanical propulsion. Unlike passive delivery systems limited by diffusion and systemic clearance, MRs offer autonomous mobility, enabling precise penetration and retention in hard-to-reach tissues. This review provides comprehensive analysis of MR technologies within urology, a field uniquely suited for microrobotic intervention due to the urinary tract’s anatomical accessibility and fluid-filled nature. We explore how MRs address critical therapeutic limitations, including the high recurrence of kidney stones and the rapid washout of intravesical bladder cancer therapies. The review categorizes propulsion mechanisms optimized for the urinary environment, such as urea-fueled nanomotors and magnetic swarms. Furthermore, we detail emerging applications, including bioresorbable acoustic robots for tumor ablation and magnetic grippers for minimally invasive biopsies. Finally, we critically assess the path toward clinical translation, focusing on challenges in biocompatibility, real-time tracking (MRI, MPI, photoacoustic imaging), and the regulatory landscape for these advanced combination products.

## 1. Introduction

The landscape of modern medicine is currently undergoing a paradigm shift, moving away from systemic pharmacotherapy, often characterized by “flooding” the body with therapeutic agents to treat localized pathologies, toward an era of precision medicine and targeted intervention [1,2,3]. Within this evolving framework, active propelled micro robots (MRs) have emerged as a transformative class of biomedical devices [4,5]. Conventional drug delivery systems (DDS), such as liposomes, polymeric nanoparticles, or monoclonal antibodies, rely predominantly on passive diffusion and systemic circulation to reach their targets [6,7]. While effective for certain applications, these passive carriers are often limited by non-specific distribution [8,9], rapid clearance by the reticuloendothelial system (RES) [10,11], and an inability to penetrate dense tissue barriers [12,13]. In contrast, MRs are engineered to actively navigate physiological environments by converting chemical [14], acoustic [15], magnetic [16], or biological energy into mechanical propulsion [17], granting them autonomous mobility.

Beyond mere locomotion, the next generation of microrobots is evolving into ‘smart’ theranostic platforms capable of sensing and responding to pathological cues. For instance, recent studies have demonstrated magnetic microrobots integrated with enzyme-responsive hydrogels that only trigger localized drug release upon exposure to tumor-associated enzymes (e.g., MMP-2/9), thereby coupling precise navigation with biologically gated actuation [18].

This comprehensive report provides an analysis of MR technologies with a specific focus on urologic diseases. The urinary tract, characterized by its anatomical accessibility via the urethra, fluid-filled lumens with water-like viscosity, and specific biochemical fuel sources (such as urea), represents arguably the most promising physiological system for the early clinical translation of these technologies. We explore how MRs address critical therapeutic limitations, including the high recurrence of kidney stones and the rapid washout of intravesical bladder cancer therapies. The review categorizes propulsion mechanisms optimized for the urinary environment, such as urea-fueled nanomotors and magnetic swarms. Furthermore, we detail emerging applications, including bioresorbable acoustic robots for tumor ablation, magnetic grippers for minimally invasive biopsies, and novel diagnostic platforms for detecting low-abundance biomarkers like TERT promoter mutations and methylated DNA. Finally, we critically assess the path toward clinical translation, focusing on challenges in biocompatibility, real-time tracking, and the regulatory landscape for these advanced combination products.

### 1.1. The Physics of Microscale Propulsion

Active propelled microrobots are miniaturized agents typically ranging in size from a few to several hundred micrometers. The defining characteristic of these agents is their ability to generate propulsive force to overcome the drag and Brownian motion inherent to fluid environments at the microscale.

To appreciate the engineering challenge that active propulsion represents, one must consider the fluid dynamics governing this regime [19,20]. Active propelled microrobots are miniaturized agents typically ranging in size from a few to several hundred micrometers. The defining characteristic of these agents is their ability to generate propulsive force to overcome the drag and Brownian motion inherent to fluid environments at the microscale [21,22,23,24].

For a swimmer of microscopic dimensions (length *L*) moving at a velocity (*v*) in a fluid of density (*ρ*) and viscosity (*μ*), the Reynolds number (*Re*) is defined as [25]:

$$Re=\frac{\rho vL}{\mu }$$

In the context of biological fluids like urine, microrobots operate in a low Reynolds number regime (*Re* << 1), often referred to as the Stokes regime [26]. In this environment, viscous forces completely dominate over inertial forces [27]. Consequently, the “coast” distance of a stopped swimmer is negligible, often less than the radius of an atom, meaning that continuous force generation is required for motion. Furthermore, the time-reversibility of Stokes flow implies that reciprocal motion, such as the simple opening and closing of a scallop’s shell, results in zero net displacement, a phenomenon famously described by Edward Purcell as the “Scallop Theorem” [28]. Therefore, effective MRs must employ non-reciprocal motion strategies, such as corkscrew rotation (mimicking bacterial flagella), traveling wave undulation, or asymmetric bubble ejection, to achieve net propulsion [29,30].

### 1.2. Classification of MRs

**Figure 1:**
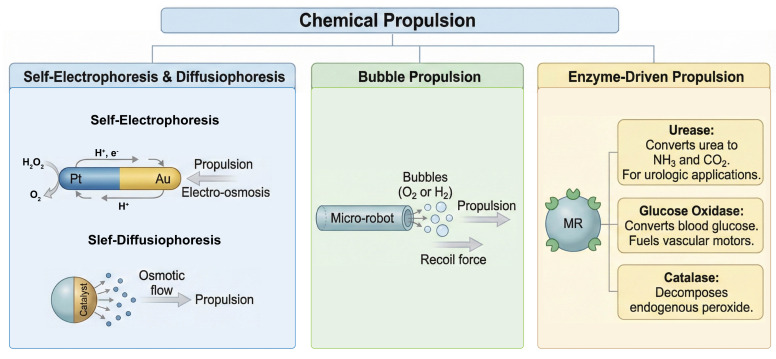
Chemical propulsion microrobots. These systems are categorized into chemically propelled, externally powered, and biohybrid types.

The classification of active MRs is primarily based on their propulsion mechanism, which dictates their design, power source, and suitability for specific in vivo applications. Broadly, they are categorized into chemically propelled, externally powered, and biohybrid systems (Figure 1).

#### 1.2.1. Chemical Propulsion MRs

Chemically propelled micromotors function as autonomous “engines,” harvesting energy from the local chemical environment by catalyzing reactions of specific fuel molecules [31,32]. These motors typically rely on asymmetric structures (Janus particles) to generate gradients that drive motion.

- Self-Electrophoresis and Diffusiophoresis MRs

Self-electrophoresis was one of the earliest mechanisms described for synthetic nanomotors. It typically involves bimetallic nanorods (e.g., Platinum-Gold) immersed in a hydrogen peroxide (H_2_O_2_) solution [33,34]. The catalytic decomposition of H_2_O_2_ occurs preferentially at the anode (e.g., Pt), generating protons (H^+^) and electrons. The electrons conduct through the metal rod to the cathode (Au), where they participate in a reduction reaction consuming protons. This flux of ions creates a local electric field around the rod. The fluid, containing charged ions, moves along the particle surface (electro-osmosis) to balance this field, resulting in the propulsion of the rod in the opposite direction [35,36]. While pioneering, the requirement for high ionic conductivity in physiological fluids often suppresses the electric double layer necessary for this mechanism, limiting its in vivo efficacy compared to other methods [37]. Specifically, the ionic strength of urine (typically 0.1–0.3 M) far exceeds the operational threshold for self-electrophoresis (~1 mM), causing the collapse of the Debye length and cessation of motion [38].

Self-diffusiophoresis is driven by the generation of a concentration gradient of solute species around the particle [39]. For instance, a Janus particle coated with a catalyst on one hemisphere will generate product molecules on that side only [40,41]. This creates a local asymmetry in solute concentration. The interaction between the solute molecules and the particle surface creates a pressure imbalance or osmotic flow that drives the particle toward regions of lower (or higher) concentration. This mechanism has been successfully employed in silica-based motors and enzyme-coated particles [42]. Unlike electrophoresis, diffusiophoresis is less sensitive to the ionic strength of the medium, making it more robust in biological fluids like urine or serum [43].

b. Bubble Propulsion MRs

Bubble propulsion represents the high-power, high-velocity end of the chemical propulsion spectrum [44,45]. This mechanism mimics jet propulsion. When a chemical reaction on the robot’s surface (often inside a tubular cavity) produces gas rapidly, bubbles nucleate, grow, and are ejected from the cavity. The momentum transfer from the ejected bubble imparts a recoil force to the robot, propelling it forward. Common fuels include hydrogen peroxide (producing O_2_ bubbles) [46] or acids reacting with metals like Zinc [47,48] or Magnesium [49,50] (producing H_2_ bubbles). These motors can achieve speeds of centimeters per second and generate sufficient force to tow cellular cargoes. However, the generation of gas bubbles in the bloodstream carries the risk of embolism, though in the urinary tract (bladder), gas release is less hazardous and potentially useful for ultrasound contrast [51].

c. Enzyme-Driven Propulsion MRs

To address the biocompatibility concerns associated with toxic metallic catalysts and fuels, enzyme-driven motors have been developed. These robots utilize biological enzymes attached to their surface to catalyze reactions using physiological fuels found abundantly in the body.

Urease-based microrobots: This enzyme converts urea (CO(NH_2_)_2_) into ammonia (NH_3_) and carbon dioxide (CO_2_). Given the high concentration of urea in urine, urease-powered motors are uniquely suited for urologic applications. They function as autonomous swimmers in the bladder or kidney without external fuel injection [52,53,54,55].

Glucose Oxidase-based microrobots: Converts blood glucose into gluconic acid and peroxide, fueling motors in the vascular system [56,57].

Catalase-based microrobots: Decomposes endogenous peroxide (present in minute amounts or produced by inflammation) to drive motion [58].

Enzyme-driven motors are inherently biocompatible and biodegradable, making them the most promising candidates for clinical translation in chemical propulsion.

#### 1.2.2. External Field Propulsion MRs

**Figure 2:**
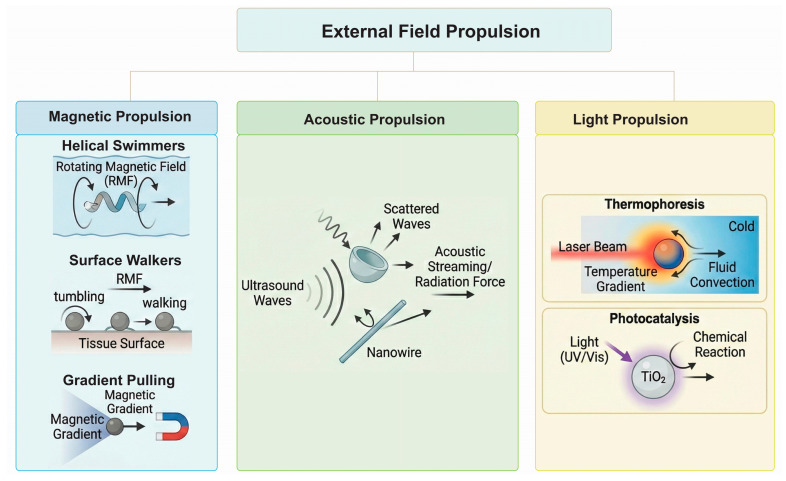
External field propulsion microrobots. Based on the external energy source, these microrobots are categorized as magnetic, acoustic, or light-driven propulsion systems.

Externally powered robots do not carry their own fuel but are driven by energy fields generated outside the patient’s body [59]. This “fuel-free” operation allows for indefinite operation time and precise external control over velocity and direction [60,61]. Based on the external energy source, these microrobots are categorized as magnetic, acoustic, or light-driven propulsion systems (Figure 2).

- Magnetic Propulsion MRs

Magnetic propulsion is the most widely investigated strategy for medical microrobots due to the transparency of human tissue to magnetic fields and the safety of low-frequency magnetic signals [62].

Helical Swimmers: Inspired by the flagella of *E. coli*, these spiral-shaped robots utilize a Rotating Magnetic Field (RMF). As the robot aligns with the rotating field, its chiral shape converts rotational motion into translational thrust via hydrodynamic coupling. This corkscrew motion is exceptionally efficient in viscous fluids and can penetrate viscoelastic media like mucus or blood clots [63,64].

Surface Walkers: Spherical or cluster-based magnetic robots can be actuated to “tumble” or “walk” along surfaces (like the bladder wall or vascular endothelium) using rotating fields. This mode exploits surface friction for traction [65,66].

Gradient Pulling: A magnetic gradient exerts a pulling force on a magnetic dipole. While simple to implement, the force scales with the volume of the magnetic material, making it weak for very small robots at a distance from the coil. Consequently, RMF actuation is generally preferred for micro-scale swimming [67,68].

b. Acoustic Propulsion MRs

Ultrasound waves can actively propel microdevices through mechanisms such as acoustic streaming or acoustic radiation force [69]. Acoustic nanomotors often feature asymmetric shapes (e.g., metallic nanowires, cup-shaped particles) that scatter sound waves unevenly [70]. This asymmetry creates a local pressure gradient or steady streaming flow that drives the particle [71]. Since ultrasound is a standard imaging and therapeutic modality in urology (e.g., lithotripsy), acoustic robots fit seamlessly into existing clinical workflows [72,73]. They can penetrate deep into tissues (kidneys) where light cannot reach and can also be used to mechanically disrupt biofilms or trigger drug release via cavitation. Critically, for drug delivery applications, these robots operate in the stable cavitation regime (oscillatory streaming) to ensure tissue safety, distinguishing them from the high-energy inertial cavitation utilized in lithotripsy to fracture stones.

c. Light Propulsion MRs

Light-driven robots utilize photonic energy to induce motion [74]. Common mechanisms include thermophoresis (where localized heating by a laser creates a temperature gradient and fluid convection) and photocatalysis (where light activates a semiconductor like TiO_2_ to drive chemical reactions) [75]. However, the penetration depth of light in tissue is limited, even in the Near-Infrared (NIR) window [76]. Therefore, light propulsion in urology is primarily envisioned for superficial applications (e.g., cutaneous lesions) or intra-organ applications where the light source is introduced endoscopically (e.g., via a cystoscope in the bladder).

#### 1.2.3. Biohybrid Propulsion MRs

**Figure 3:**
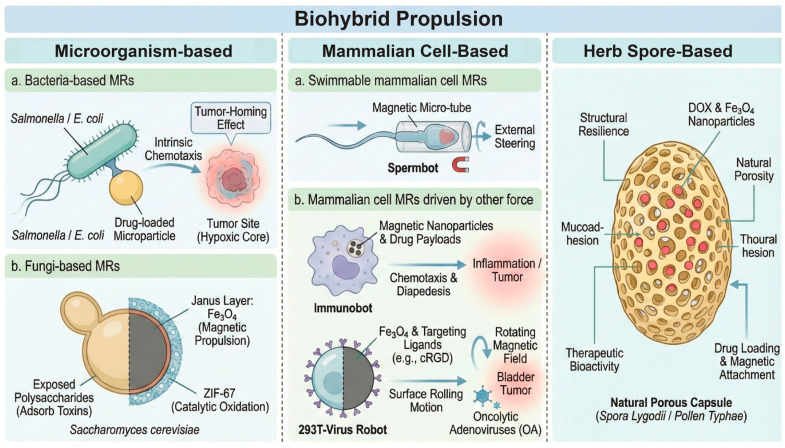
Biohybrid propulsion microrobots. These microrobots are categorized as microorganism-based, mammalian cell-based, and herb-based systems.

Biohybrid systems combine synthetic functional components (drug payloads, magnetic caps) with biological actuators (live cells or microorganisms) (Figure 3) [77]. This approach harnesses millions of years of evolutionary optimization in biological motility and sensing.

- Microorganism-based MRs

i. Bacteria-based MRs

Bacteria such as *Salmonella typhimurium* or *E. coli* are attached to drug-loaded microparticles. These bacteria possess intrinsic chemotactic abilities, allowing them to sense and swim toward specific chemical gradients [78]. A critical advantage in oncology is the tumor-homing effect: anaerobic bacteria naturally congregate in the hypoxic necrotic cores of tumors, areas that are often inaccessible to blood-borne drugs. By hitching a ride on these bacteria, microrobots can be autonomously guided to the most treatment-resistant parts of a tumor. While biofouling is a major hurdle in vascular transport, the urinary environment, lacking high protein and cellular density, naturally mitigates non-specific aggregation for these bacterial swimmers, though surface modifications (e.g., cell membrane camouflaging) remain an option to prevent immune clearance [79].

ii. Fungi-based MRs

Yeast cells (*Saccharomyces cerevisiae*) offer a robust, thick-walled chassis ideally suited for environmental detoxification, a capability that has been advanced through the development of Janus Yeast Cell robots (JYC-robots). Fabricated via an asymmetric masking technique, these biohybrid systems feature a sophisticated dual-function coating on one hemisphere of the cell. The architecture consists of an S. cerevisiae core partially coated with Fe_3_O_4_ nanoparticles to enable magnetic propulsion, followed by the in situ growth of Zeolitic Imidazolate Framework-67 (ZIF-67), a cobalt-based metal–organic framework, directly onto the magnetic patch. This strategic Janus configuration is critical to the robot’s function: it ensures magnetic mobility while leaving the opposing hemisphere of the native cell wall exposed. The uncoated cell wall, rich in polysaccharides such as glucans and mannans, naturally adsorbs mycotoxins like Zearalenone (ZEN), while the ZIF-67 layer contributes additional porosity and catalytic oxidation capabilities, resulting in a synergistic platform for efficient toxin removal [80].

Beyond simple coating strategies, advanced fabrication techniques have produced magnetite nanostructured porous hollow helical microswimmers, which offer high surface area for drug loading and efficient magnetic actuation [81]. Furthermore, multifunctional biohybrid robots derived from Spirulina have been engineered to degrade on command while providing contrast for imaging-guided therapy, establishing a benchmark for biocompatible theranostics [82].

b. Mammalian Cell-Based MRs

i. Swimmable mammalian cell MRs

Sperm cells are powerful natural swimmers adapted to navigate the female reproductive tract, which shares rheological similarities with other viscous bodily fluids. “Spermbots” involve trapping a single sperm cell inside a magnetic micro-tube or coating the sperm head with magnetic nanoparticles [83]. The sperm provides the high-velocity flagellar propulsion and the ability to penetrate mucus, while the synthetic magnetic component allows for external steering. These hybrids are biocompatible and can protect drugs from immune clearance, making them candidates for gynecological and urologic drug delivery.

ii. Mammalian cell MRs driven by other forces

(1) Immunobots: Immune cells like macrophages and neutrophils can be engineered to internalize magnetic nanoparticles and drug payloads. These “immunobots” leverage the natural ability of immune cells to migrate toward inflammation (chemotaxis) and cross biological barriers (diapedesis). Recent innovations have demonstrated macrophage-based microrobots (MΦ-OMV robots) engineered with bacterial outer membrane vesicles (OMVs). These robots utilize magnetic propulsion to accumulate at tumor sites, where they deploy a multi-modal attack: tumor inhibition via macrophage activity, immune regulation via OMVs, and the release of anticancer peptides [84,85].

(2) 293T and tumor cell MRs: For larger eukaryotic cells, solid-phase synthesis is employed to create “Janus” cell robots. A notable advancement in this area is the 293T-Virus Robot. This system utilizes human embryonic kidney (293T) cells as a chassis to load and replicate oncolytic adenoviruses (OA). The cells are asymmetrically coated with magnetic nanoparticles and targeting ligands (e.g., cRGD). Under a rotating magnetic field, these robots exhibit a “surface rolling” motion that enhances tissue retention and penetration. Once at the target site (e.g., bladder tumor), the robot releases the oncolytic virus, which infects and destroys cancer cells while sparing normal tissue. This approach addresses the limitations of passive viral therapy, such as rapid washout and poor tumor penetration [80,86].

c. Herb Spore-Based Microrobots: Natural Porous Capsules

Moving beyond mammalian and microbial lineages, plant spores and pollen grains present a unique chassis for microrobotic design, distinguished by their exceptional structural resilience and intrinsic natural porosity. Notable examples include Spora Lygodii (SL), derived from the fern Lygodium japonicum, and Pollen Typhae (PT) from Typha species. SL is particularly relevant for urologic applications as it is a traditional Chinese medicinal material used for treating urinary dysfunctions; its fabrication involves a dip-coating process where chemotherapeutics like Doxorubicin (DOX) and Fe_3_O_4_ nanoparticles are sequestered within the natural microchannels of the sporoderm. Similarly, PT grains utilize their uniform size (~20 μm) and reticulated exine structure to provide a high surface area for the attachment of magnetic propulsion elements and drug payloads. The defining advantage of these spore-based systems is their non-living nature, which eliminates the logistical burden of maintaining cell viability during storage and transport, yet they retain critical “bio-advantages” such as natural mucoadhesion and intrinsic therapeutic bioactivities, including hemostasis and litholytic properties [62,87].

A comparative summary of these propulsion mechanisms, detailing their speed, safety, and specific suitability for urologic environments, is provided in Table 1.

## 2. Urologic Disease Burden and Urology System: A Perfect System for MRs

### 2.1. The Clinical Imperative in Urology

**Figure 4:**
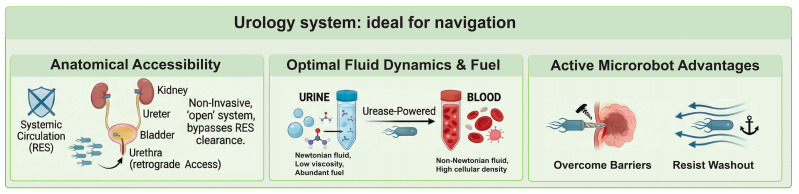
The favorable anatomical environment for microrobotic interventions in urology. The diagram illustrates the unique advantages the urinary tract offers for microrobot deployment.

The burden of urologic diseases on global healthcare systems is substantial and accelerating. Driven by aging populations, dietary shifts, and environmental factors, urologic pathologies represent a growing crisis in morbidity and healthcare economics [88,89,90,91]. While traditional surgical and pharmacological interventions have advanced, they face fundamental physical limitations, specifically regarding drug retention, tissue penetration, and the complete elimination of disease foci (Figure 4).

Urolithiasis, or kidney stone disease, affects approximately 12% of the global population at some point in their lives, with China identified as one of the three major high-incidence regions [92,93]. The condition is painful and costly, but the most significant challenge is the high recurrence rate, up to 50% of patients will form another stone within 5–10 years, a trend consistent even in high-incidence populations like China [94]. Current treatments like Extracorporeal Shock Wave Lithotripsy (SWL) and Ureteroscopy (URS) are effective at breaking stones but often leave behind “residual fragments” or dust [95]. These micro-fragments serve as a nidus for rapid stone regrowth, perpetuating the cycle of disease.

Bladder cancer presents an even more daunting challenge. It is the 10th most common cancer globally and the 4th most common in men [96,97]. It is characterized by the highest recurrence rate of any malignancy, reaching up to 70–80% for Non-Muscle Invasive Bladder Cancer (NMIBC) [98,99]. This necessitates lifelong surveillance via cystoscopy and repeated treatments, making bladder cancer one of the most expensive cancers to treat per patient [100]. The standard adjuvant therapy, intravesical instillation of BCG (Bacillus Calmette-Guérin) or chemotherapy (e.g., mitomycin C, gemcitabine), suffers from poor efficacy because the bladder constantly produces urine that dilutes the drug and washes it out during voiding [101,102]. This “washout effect” limits the residence time of the drug at the tumor site. Furthermore, the bladder urothelium is a highly impermeable barrier [103], designed to protect the body from urine toxins, which also prevents the penetration of therapeutic agents into the bladder wall where tumors reside.

For Upper Tract Urothelial Carcinoma (UTUC) located in the ureter or renal pelvis, the situation is even more complex [104]. Topical treatment is difficult because urine flow and ureteral peristalsis constantly wash drugs downstream into the bladder. Systemic chemotherapy is often required, which carries significant toxicity risks for the kidneys and other organs [105]. Thus, there is an urgent unmet clinical need for technologies that can actively resist urine flow, penetrate tissue barriers, and deliver concentrated payloads directly to the site of pathology.

### 2.2. Urology System Is a Perfect System for Microrobots Navigation

While the potential of medical microrobotics has been explored across various organ systems, the urinary tract presents a unique confluence of anatomical accessibility, fluid dynamics, and metabolic resources that arguably make it the most viable environment for early clinical translation. Unlike the brain or cardiovascular system, which require highly invasive catheterization through the tortuous vascular tree and are guarded by tight endothelial barriers, the urinary tract is an “open” anatomical system. This unique architecture allows for retrograde access via the urethra, enabling the non-invasive deployment of microrobotic swarms directly into the bladder, ureters, or kidneys while completely bypassing the systemic circulation. This approach effectively circumvents the Reticuloendothelial System (RES) clearance that typically sequesters intravenous nanomedicine in the liver and spleen [106]. Once introduced, microrobots encounter a hydrodynamic environment that is ideal for locomotion; urine is a Newtonian fluid with a density of approximately 1 g/cc and dynamic viscosity of 0.01 g/(cm/s) [107]. This is nearly identical to water and stands in stark contrast to blood, which acts as a non-Newtonian, shear-thickening fluid with high cellular density. The absence of hematocrit in urine significantly reduces drag forces and minimizes the risk of bio-fouling or non-specific aggregation, thereby simplifying the low-Reynolds-number physics required for propulsion. Furthermore, the urinary tract provides an indigenous chemical fuel source for autonomous operation: urea. As an abundant metabolic waste product, urea can be harvested by urease-functionalized nanomotors to generate propulsion indefinitely. This offers a distinct safety advantage over glucose-driven motors in the blood, where fuel depletion could be metabolically dangerous. When coupled with the ability to image these agents using standard urological ultrasound and the natural capacity to void the robots after the mission is complete, the urinary tract emerges not just as a target for therapy, but as the perfect physiological playground for the next generation of active biomedical machines (Figure 4).

The transition from passive diffusion to active robotic delivery offers critical advantages, primarily in the ability to overcome the potent physical barriers that define urologic pathologies. High-grade bladder cancer and renal cell carcinoma are often shielded by a dense extracellular matrix and elevated interstitial fluid pressure, which severely limit the penetration of passively diffusing chemotherapeutics [108,109]. Active microrobots, however, can generate piconewton to nanonewton forces, enabling them to mechanically burrow into tumor tissue or penetrate the highly impermeable urothelium, significantly increasing the bioavailability of therapeutic payloads [110,111]. Furthermore, the urinary tract is a dynamically active system, characterized by fluid flows ranging from the peristaltic pumping of the ureters to the forceful voiding of the bladder. While passive particles are susceptible to rapid convective washout, active microrobots can surmount these forces by exhibiting rheotaxis (upstream swimming) or utilizing active anchoring mechanisms. This capability allows them to maintain position against physiological flow, ensuring sustained drug release at the target site rather than being flushed away before therapeutic efficacy is achieved [80].

Finally, the integration of microrobotics into urology introduces a level of precision and multifunctionality unattainable with conventional tools. Through responsiveness to external steering fields (magnetic control) or internal chemical gradients (chemotaxis), microrobots can be guided to specific focal lesions with high spatial resolution [86,87]. This is crucial for reaching hard-to-access anatomical locations, such as a specific lower renal calyx containing a stone fragment or a localized upper tract tumor, while sparing healthy tissue from exposure to toxic agents. Beyond simple targeting, these agents function as versatile micro-tools capable of complex “theranostic” tasks. They can be engineered to perform mechanical ablation, such as scrubbing bacterial biofilms from indwelling stents, or chemical modulation, such as elevating local pH to dissolve uric acid stones [112,113,114]. Additionally, they can serve as micro-grippers for diagnostic biopsy or carriers for imaging contrast agents, effectively closing the loop between diagnosis and therapy in a single, minimally invasive procedure [115,116].

### 2.3. Applications of Micro Robots in Urologic Disease

The application of MRs in urology addresses specific limitations of current therapies: poor retention, lack of penetration, and inability to target specific focal lesions.

#### Bladder Disease

- Applications of Microrobots in Bladder Cancer

**Figure 5:**
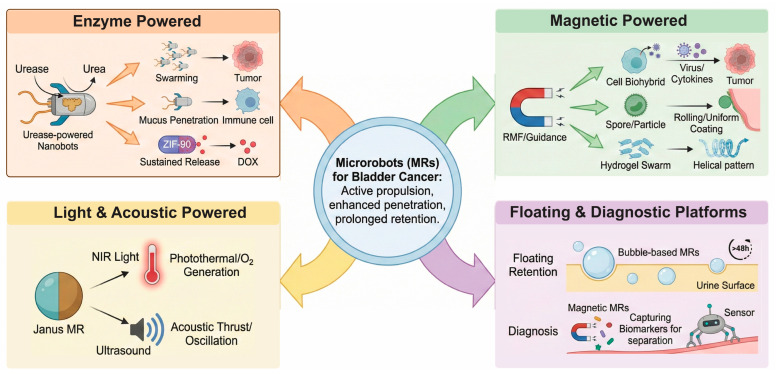
Applications of microrobots in bladder cancer. Various propulsion strategies are employed to improve tissue penetration and intravesical retention, addressing key limitations in current treatments.

Bladder cancer (BC) management faces significant challenges, primarily due to the high recurrence rates of non-muscle-invasive bladder cancer (NMIBC) and the limited residence time of intravesical therapeutics caused by periodic urination. Microrobots MRs offer a transformative solution by providing active propulsion, enhanced tissue penetration, and prolonged retention within the bladder cavity (Figure 5) [117,118].

i. Enzyme-Powered Nanobots: Harnessing Endogenous Fuels

Urease-powered nanobots utilize the high concentration of urea present in the bladder as a chemical fuel to generate self-propulsion, enhancing the delivery of various therapeutic payloads.

Radionuclide Therapy: Recent studies have demonstrated the use of urease-powered mesoporous silica nanobots labeled with Iodine-131. These nanobots exhibit swarming behavior that enhances fluid mixing and prevents sedimentation, resulting in an 8-fold increase in tumor accumulation compared to passive particles and a approx. 90% reduction in tumor volume in orthotopic mouse models [117].

Immunotherapy Activation: To overcome the mucus barrier, urease-powered nanomotors based on chitosan/heparin nanocomplexes have been developed to deliver STING agonists. These motors penetrate the bladder mucus layer and activate the STING pathway, recruiting CD8+ T cells and inhibiting tumor growth by 94.2% [117].

Anti-Biopassivation Strategies: A critical limitation of enzymatic motors is the rapid deactivation of enzymes in physiological fluids. To address this, “Ur-MOFtors” were engineered by embedding urease within Zeolitic Imidazolate Framework-90 (ZIF-90). This reticular structure protects the enzyme via hydrogen bonding with catalytic products, extending the locomotion lifespan to over 90 min (an 18-fold enhancement) and enabling effective delivery of Doxorubicin [119].

ii. Magnetic-Actuated Systems: Precision Guidance

Magnetic actuation allows for fuel-free, precise navigation of microrobots to tumor sites, often combined with biological carriers or hydrogels.

Cell-Based Biohybrids:

Oncolytic Virotherapy: A “Trojan horse” strategy has been designed using 293T cells infected with oncolytic adenovirus (OA). Coated with magnetic nanoparticles and cRGD peptides, these cell robots actively target αvβ3 integrin on cancer cells, releasing the virus to induce cytolysis [80].

Macrophage Immunobots: Macrophages engineered to engulf magnetic nanoparticles and bacterial outer membrane vesicles (OMVs) serve as chemotactic robots. These robots leverage the natural tumor tropism of macrophages and the immune-stimulating properties of OMVs for multimodal therapy [85]. Similarly, macrophage-based “immunobots” carrying magnetic decoy bacteria have been shown to polarize towards an anti-tumor M1 phenotype, secreting cytokines (TNF-α, IL-6) to kill bladder cancer cells [120].

Natural Spore Carriers: The porous spores of Traditional Chinese Medicine (TCM) *Spora Lygodii* have been utilized as magnetic carriers for Doxorubicin. These microrobots (Fe/DOX@SL) exhibit rolling motion under a rotating magnetic field, enhancing mucosal adherence and tumor suppression [87].

Hydrogel Swarms: Magnetic hydrogel robots (MHRMs) composed of sodium alginate and NdFeB nanoparticles have been developed to deliver Mitomycin C. These robots perform a helical motion to uniformly coat the bladder wall with the drug, addressing the issue of uneven drug distribution caused by gravity [118].

iii. Light and Acoustic-Driven Systems

External stimuli such as light and ultrasound offer alternative propulsion mechanisms that can also induce therapeutic effects like hyperthermia. Photosynthetic Biohybrids: Light-driven microrobots based on the algae *Chlamydomonas reinhardtii* conjugated with glycol chitosan-polypyrrole (GCS-PPy) nanoparticles combine phototaxis (active movement toward light) with photosynthesis. Upon irradiation, they generate oxygen to alleviate tumor hypoxia and produce heat (photothermal therapy), effectively modulating the tumor microenvironment to favor anti-tumor immunity [121]. Photothermal Janus Motors: Janus micromotors fabricated from Black TiO_2_ and Gold (Au) utilize urease for propulsion and Near-Infrared (NIR) light for photothermal therapy. These motors demonstrate high velocity in urine (30.28 μm/s)and effective cancer cell ablation under laser irradiation [55]. Acoustic Hydrogels: Bioresorbable Acoustic Microrobots (BAMs) featuring air-trapping cavities oscillate under ultrasound to generate thrust. These hydrogel robots are designed with a hydrophobic inner surface to retain bubbles for days, allowing for long-term ultrasound imaging and magnetically guided chemotherapy delivery [122].

iv. Floating Retention and Diagnostic Platforms

Innovative designs also focus on passive retention strategies and diagnostic capabilities. Floating Delivery Systems: To counter the rapid voiding of drugs during urination, BCG-loaded air microbubbles (BCG-MBs) have been developed. Their low density (509 kg/m^3^) allows them to float in the urine, achieving retention times exceeding 48 h and inducing durable immune responses [123]. Diagnostic Nanomotors: Urease-driven Janus magnetic nanomotors (Ur-JMNMs) functionalized with hairpin DNA have been engineered for the multiplexed detection of bladder cancer biomarkers (miRNA-21 and miRNA-182) in urine. The active motion accelerates target capture, while magnetic separation enhances detection sensitivity [54]. Mechanical Characterization: For transurethral diagnostics, a continuum robot manipulator equipped with a microforce sensor has been proposed to palpate the bladder wall, identifying tumorous tissue via viscoelastic property characterization [124].

v. Interstitial Cystitis/Bladder Pain Syndrome (IC/BPS)

To address the chronic inflammation and delivery challenges associated with Interstitial Cystitis/Bladder Pain Syndrome (IC/BPS), researchers have developed innovative microrobotic platforms capable of penetrating the bladder’s protective mucus layer. One approach utilizes soft “microgelbots,” composed of magnetic nanochains embedded within a poly(ethylene glycol) diacrylate (PEGDA) microgel. These robots are optimized with a quadrangle shape to rotate under a magnetic field, generating sufficient shear force to actively penetrate the shear-thinning mucus layer. When loaded with mesenchymal stem cells (MSCs), these microgelbots demonstrated prolonged retention in the bladder and successfully inhibited mast cell infiltration, collagen deposition, and cell apoptosis in a cyclophosphamide-induced IC mouse model via paracrine effects [125]. Another strategy involves acoustic bubble-based microrobots designed with asymmetric fins and an air-trapping cavity. Upon excitation by ultrasound, the trapped air bubble oscillates to drive the robot at high speeds of approximately 150 body lengths per second, while the asymmetric fins induce orbital motion and allow the device to mechanically “pin” itself into the soft bladder epithelium. This mechanical anchoring facilitates the sustained release of dexamethasone over several days, effectively polarizing macrophages from a pro-inflammatory (M1) to an anti-inflammatory (M2) phenotype to temper the immune response [45].

vi. Underactive Bladder (UAB)

For patients suffering from Underactive Bladder (UAB), where reduced detrusor muscle contraction prevents effective voiding, a macro-scale magnetic soft robotic system known as the Intelligent Bladder Volume Control System (IBCS) has been proposed to restore voluntary urination. This system integrates an implantable “Meshed Magnetic Soft Robot” (MMR) that is sutured directly to the bladder wall with a wearable external magnetic sensor. The sensor continuously monitors bladder volume by detecting changes in magnetic flux generated by the MMR as the bladder expands. When the bladder reaches capacity, an external magnetic field actuates the MMR, causing it to mechanically compress the bladder. In trials with a UAB pig model, this system generated intravesical pressures up to 33 cmH_2_O and achieved a voiding efficiency of greater than 83%, demonstrating its potential as a functional replacement for the detrusor muscle [126].

b. Applications of MRs in Kidney Disease

**Figure 6:**
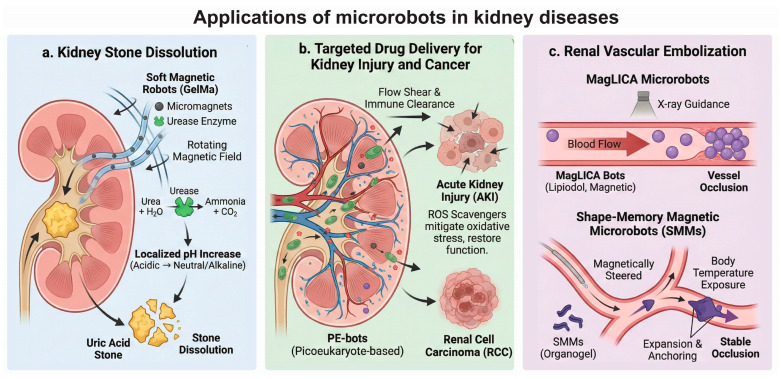
Applications of microrobots in kidney diseases. The applications include non-invasive stone dissolution via urease-based pH modulation, targeted drug delivery for acute kidney injury and cancer, and precise vascular embolization using magnetic or shape-memory microrobots.

MRs offer transformative solutions for renal pathologies by navigating the kidney’s complex anatomical and physiological barriers. Leveraging active propulsion and functional materials, these systems provide precise, minimally invasive alternatives to conventional therapies. The following subsections detail their application in stone dissolution, targeted drug delivery, and vascular embolization (Figure 6).

i. Kidney Stone Dissolution

To address the high prevalence and recurrence of kidney stones, particularly uric acid stones, a non-invasive therapeutic strategy using enzyme-loaded soft magnetic robots has been developed. These millimeter-scale robots consist of flexible gelatin methacryloyl (GelMa) filaments embedded with micromagnets for navigation and loaded with the enzyme urease to trigger localized chemical changes. Under the control of a rotating magnetic field, these robots can be navigated through the urinary tract into the renal pelvis, where the embedded urease catalyzes the hydrolysis of urea found in urine into ammonia and carbon dioxide. This reaction locally increases the urinary pH from acidic levels (around 5.6) to neutral or slightly alkaline levels (around 7.0–7.2), creating an environment favorable for the dissolution of uric acid stones. In vitro experiments utilizing a 3D-printed human urinary tract model demonstrated that these robots could reduce the mass of uric acid stones by approximately 30% within 5 days, offering a potentially catheter-free or minimally invasive alternative to surgical lithotripsy for stone management [113].

ii. Targeted Drug Delivery for Kidney Injury and Cancer

Navigating the complex and confined microenvironments of the kidney for precise drug delivery remains a significant challenge, which has been addressed by the development of picoeukaryote-based biohybrid microrobots (PE-bots). These microrobots are engineered from the unicellular algae *Ostreococcus tauri*, functionalized with superparamagnetic Fe_3_O_4_ nanoparticles for magnetic propulsion and therapeutic agents for specific disease targets. Due to their ultrasmall size and biological flexibility, PE-bots can effectively navigate through the renal vasculature and interstitial spaces, overcoming physiological barriers such as flow shear and immune clearance. In murine models, these robots demonstrated exceptional kidney-targeting capabilities with retention times exceeding 48 h. For the treatment of Acute Kidney Injury (AKI), PE-bots loaded with reactive oxygen species (ROS) scavengers successfully mitigated oxidative stress and restored renal function. Furthermore, when loaded with the chemotherapeutic agent Doxorubicin (DOX), they exhibited targeted inhibition of Renal Cell Carcinoma (RCC), significantly reducing tumor burden while minimizing systemic toxicity compared to passive drug carriers [53].

iii. Renal Vascular Embolization

Vascular embolization is a critical intervention for treating renal conditions such as tumors or hemorrhages by occluding blood flow, and recent advancements in microrobotics have enhanced the precision and stability of this procedure. One approach utilizes magnetically responsive lipiodol-calcium alginate (MagLICA) microrobots, fabricated using an intelligent high-throughput oscillatory shear technology. These microrobots possess high sphericity and magnetic responsiveness, allowing them to be navigated through blood vessels against fluid flows of up to 4 mL/min under real-time X-ray guidance due to the radiopacity of Lipiodol. They have proven effective in accurately embolizing kidney blood vessels in vivo, addressing the limitations of catheter accessibility in tortuous vessels [127]. Another strategy involves shape-memory magnetic microrobots (SMMs) composed of a magnetic particle-embedded organogel. These robots are delivered via a catheter to vessel bifurcations and then magnetically steered to the target renal artery. Upon exposure to body temperature, the SMMs undergo a shape-memory recovery expansion, anchoring themselves securely within the vessel lumen to resist blood flow and induce stable occlusion, thereby effectively blocking blood supply to the target renal tissue [128].

c. Applications of Microrobots in Prostate Cancer

**Figure 7:**
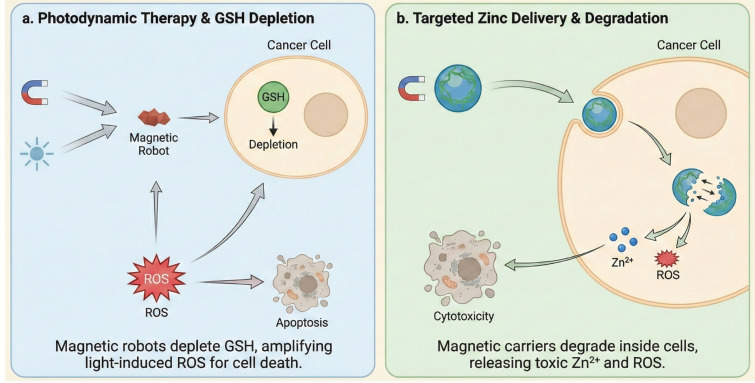
Applications of microrobots in prostate cancer.

Microrobots are being developed to address therapeutic challenges in prostate cancer by targeting its unique tumor microenvironment. These approaches include using multi-propulsive robots to enhance photodynamic therapy via antioxidant depletion and employing biodegradable carriers to exploit metabolic vulnerabilities through targeted zinc delivery (Figure 7).

i. Photodynamic Therapy and GSH Depletion

To address the limitations of photodynamic therapy (PDT) in the hypoxic and antioxidant-rich tumor microenvironment, self-propelled magnetic dendrite-shaped microrobots based on hematite (α-Fe_2_O_3_) have been developed. These microrobots feature a unique dual-propulsion capability: they exhibit negative phototactic motion under light irradiation and can be precisely navigated along defined paths using an external magnetic field. A critical barrier to effective PDT is the overexpression of glutathione (GSH) in tumor cells, which scavenges the Reactive Oxygen Species (ROS) generated during therapy. These hematite microrobots actively deplete intracellular GSH through a redox reaction that reduces hematite to ferrous ions (Fe^2+^), thereby disrupting the tumor’s antioxidant defense. This GSH depletion significantly amplifies the oxidative stress induced by PDT, leading to enhanced apoptosis in prostate cancer cells (PC-3). Furthermore, the dendritic morphology facilitates the non-contact transportation of cells via hydrodynamic effects, offering a multifunctional platform for targeted cancer treatment [129].

ii. Targeted Zinc Delivery via Self-Degrading Carriers

Prostate cancer cells are metabolically distinct in their inability to accumulate zinc, a mineral that is typically cytotoxic to them at high concentrations. Leveraging this vulnerability, biodegradable magnetic microrobots composed of the amino acid cystine and zinc ions have been engineered for targeted therapy. These robots encapsulate superparamagnetic iron oxide nanoparticles (Fe_3_O_4_ NPs), allowing for precise navigation under a rotating magnetic field. Their therapeutic mechanism relies on the specific reducing environment of cancer cells, characterized by high levels of GSH and NADH. Upon internalization by prostate cancer cells, the disulfide bonds of the cystine matrix and the non-covalent metal-ligand interactions are cleaved by the intracellular reducing agents. This triggers the self-disassembly of the robot and the subsequent release of zinc ions. The intracellular accumulation of zinc, combined with the generation of ROS during degradation, induces significant cytotoxicity and apoptosis in prostate cancer cells, offering a site-specific and bio-responsive therapeutic strategy [47].

d. Urinary Tract Infections (UTIs) and Biofilms

In the context of persistent urinary tract infections (UTIs) and catheter-associated infections caused by bacterial biofilms, particularly *Escherichia coli*, hybrid enzyme-photocatalyst tandem microrobots (U-urobots) offer a potent solution. These nanobots are constructed from TiO_2_/CDS nanotube bundles functionalized with urease, allowing them to operate via a dual mechanism. The urease enzyme enables self-propulsion using urea, which is naturally present in urine, as a fuel source, while the TiO_2_/CDS structure acts as a photocatalyst under visible light. The synergy of active motion, which enhances penetration into the dense biofilm matrix, and light activation, which generates cytotoxic Reactive Oxygen Species (ROS), resulted in the removal of nearly 90% of *E. coli* biofilms in vitro, significantly outperforming passive or dark control groups [130]. Additionally, bioadhesive bacteriabots have been developed by attaching motile *E. coli* bacteria to functionalized microparticles. These robots utilize the natural affinity of bacterial type 1 pili (lectin) for mannose molecules expressed on bladder epithelial cells to anchor themselves to the target tissue, thereby facilitating targeted drug delivery and retention [131].

e. Erectile Dysfunction

In the realm of sexual health, the treatment of Erectile Dysfunction (ED) has seen significant advancement through the development of ultrasoft, shape-adaptive microrobots designed to overcome the physiological barriers of the corpus cavernosum. To address the poor retention and survival of therapeutic cells in this highly vascularized tissue, researchers engineered “MSC-Robs”, magnetic hydrogel microrobots encapsulating Mesenchymal Stromal Cells (MSCs). These robots are composed of a polyethylene glycol (PEG) matrix embedded with magnetic nanoparticle chains for navigation and functionalized with phenylboronic acid to scavenge excessive Reactive Oxygen Species (ROS), a key contributor to ED pathology. Their ultrasoft mechanical property (Young’s modulus < 1 kPa) allows them to deform and navigate through narrow microvessels without causing occlusion, ensuring a uniform distribution of the therapeutic payload. In preclinical models, these robots demonstrated superior retention compared to free cell injections and successfully promoted angiogenesis, neurogenesis, and smooth muscle regeneration, ultimately restoring erectile function and fertility [132].

### 2.4. Imaging and Tracking Strategies for Microrobots in Urologic Disease

**Figure 8:**
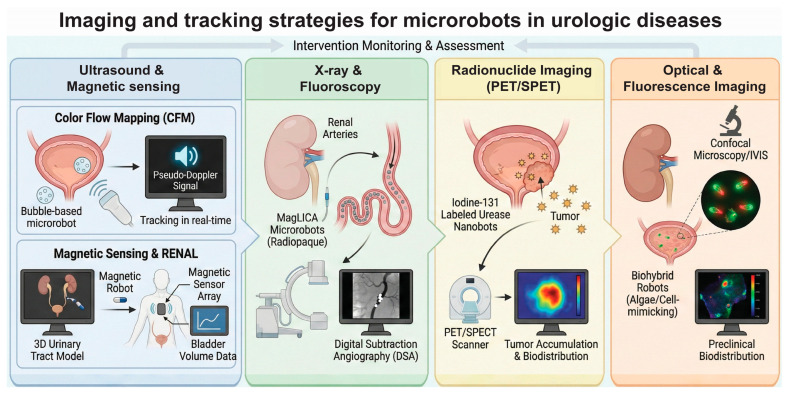
Imaging and tracking strategies for microrobots in urologic diseases. A range of modalities supports real-time monitoring, such as ultrasound and magnetic sensing for navigation, X-ray for vascular tracking, and radionuclide or optical imaging for evaluating biodistribution and tumor accumulation.

Effective clinical translation of microrobotic interventions requires precise, real-time imaging to monitor navigation, verify localization, and assess therapeutic efficacy in deep tissues. A spectrum of modalities enables this real-time monitoring, each addressing specific physiological barriers. Ultrasound and magnetic sensing are primarily utilized for active navigation, offering deep tissue penetration and closed-loop feedback control essential for maneuvering within the urinary tract. For vascular applications, X-ray fluoroscopy provides high temporal resolution and deep penetration, allowing for the precise tracking of radiopaque microrobots during embolization procedures. Furthermore, evaluating therapeutic efficacy requires quantitative tracking; radionuclide imaging (such as PET or SPECT) is employed to assess whole-body biodistribution, while optical imaging offers high-sensitivity validation of tumor accumulation, particularly in preclinical models (Figure 8).

Ultrasound (US) imaging has emerged as a leading modality due to its safety and deep penetration. A significant advancement in this domain is the application of Color Flow Mapping (CFM) to visualize bubble-based microrobots. Unlike standard B-mode imaging which struggles with microscale resolution, CFM detects the “pseudo-Doppler” signals generated by the acoustic oscillations of trapped air bubbles within the robots. This technique allows for the real-time identification and tracking of individual microrobots against the background of static tissue, as demonstrated in ex vivo mouse bladders [133]. Furthermore, ultrasound has been integrated into navigation frameworks like RENAL, where it is used to reconstruct 3D urinary tract models and track magnetic robots in real-time, enabling pathfinding algorithms to guide them through complex anatomies like the renal pelvis [134]. In the context of bladder volume monitoring, ultrasound is complemented by magnetic sensing technologies. For instance, a wearable magnetic field sensor array was developed to continuously track the expansion of a magnetic soft robot sutured to the bladder wall, translating magnetic flux changes into real-time bladder volume data to manage underactive bladder [126].

For applications requiring high-contrast visualization of the vascular system, such as renal embolization, X-ray and Fluoroscopy are utilized. Intelligent fabrication methods (iGHOST) have produced alginate-based microrobots (MagLICA) incorporated with Lipiodol, a radiopaque oil. This integration renders the microrobots highly visible under X-ray Digital Subtraction Angiography (DSA), allowing clinicians to navigate them magnetically through tortuous renal arteries and visually confirm vascular occlusion in real-time [127]. Radionuclide imaging represents another powerful strategy, particularly for theranostics. Urease-powered nanobots labeled with Iodine-131 enable dual functionality: they deliver targeted radiotherapy to bladder tumors while simultaneously allowing for identifying their accumulation and biodistribution via Positron Emission Tomography (PET) or Single Photon Emission Computed Tomography (SPECT) [128]. Finally, optical and fluorescence imaging (e.g., IVIS, confocal microscopy) remain critical for preclinical validation. These modalities are extensively used to track the biodistribution of biohybrid robots, such as algae-based swimmers or cell-mimicking carriers, confirming their retention in the kidney or bladder tissues over time, although their limited penetration depth restricts their use in human clinical settings [47,53].

## 3. Clinical Translation: Challenges and Outlook

Translating microrobotic technologies from preclinical models to human urologic applications presents a complex set of challenges that starkly contrasts with the field’s rapid academic progress. Despite over two decades of innovation in propulsion and design, the transition from laboratory proof-of-concept to clinical utility remains stalled in the “Valley of Death.” To bridge this gap, the field must shift focus from novel swimming gaits to addressing the rigid constraints of clinical safety, deep-tissue actuation, and real-time visualization, effectively advancing from low Technology Readiness Levels (TRL) to systems capable of integration into standard medical workflows [135].

### 3.1. Biocompatibility and Safety Hurdles

A primary barrier to clinical adoption is long-term biocompatibility. While materials like polyethylene glycol (PEG), alginate, and iron oxide nanoparticles (IONPs) are generally considered safe, their behavior in the dynamic microenvironment of the human body requires rigorous scrutiny. IONPs, the gold standard for magnetic actuation, risk accumulation in the reticuloendothelial system (liver and spleen) if not properly cleared [136]. Furthermore, the degradation products of synthetic microrobots must be non-toxic and excretable. The challenge is even more acute for biohybrid systems that utilize bacteria or sperm for propulsion. While effective in vitro, bacterial-based robots carry significant risks of immunogenicity. They may trigger adverse immune reactions or be prematurely cleared by the host’s defense mechanisms before reaching their therapeutic targets. Conversely, in bladder cancer immunotherapy, this immune stimulation could be harnessed synergistically, but controlling the magnitude of the response to prevent sepsis is a critical safety threshold [137].

### 3.2. The Barriers of Actuation and Visualization

Controlling microrobots deep within the human body, such as in the kidney or prostate, presents significant engineering hurdles. Magnetic actuation, the most promising modality, faces the physical reality that magnetic field gradients attenuate rapidly over distance (1/r^4^). Generating sufficient force to navigate against blood flow or urine currents in deep tissue requires massive external coils or robotic arm-mounted permanent magnets that are compatible with the sterile operating room environment [138].

However, the single greatest bottleneck is visualization. If a clinician cannot see the robot, they cannot control it. Conventional imaging modalities fall short: MRI is too slow for real-time tracking, X-ray fluoroscopy involves ionizing radiation, and standard B-mode ultrasound lacks the resolution to distinguish microrobots from tissue speckle [139]. Recent breakthroughs in Color Flow Mapping (CFM) ultrasound and photoacoustic imaging offer a solution. By detecting the “twinkling artifacts” or pseudo-Doppler signals generated by oscillating microrobots, clinicians may soon be able to track individual agents in real-time without radiation, a critical step for closed-loop navigation [133].

### 3.3. Future Outlook

Despite these challenges, the outlook for urologic microrobotics is optimistic, driven by a pragmatic shift toward “macro–micro” hybrid systems. Rather than swarms of invisible nanobots, the immediate future lies in tetherless millirobots, visible, retrievable devices that can be deployed via catheters. A prime example is the development of urease-loaded magnetic polymer strips for kidney stone treatment. These macroscopic agents can be magnetically guided into the renal pelvis to chemically dissolve uric acid stones, offering a non-invasive alternative to surgery while ensuring the device can be fully retrieved post-treatment [113].

In bladder cancer, future platforms will likely focus on active retention to overcome the “washout” effect of urine voiding. Microrobots equipped with mucoadhesive anchors or those that can burrow into the bladder wall (e.g., acoustic bubble-propelled micro-rockets) promise to extend drug residence time from hours to days [109]. Furthermore, the field is expanding into regenerative medicine. Soft, shape-adaptive microrobots are being engineered to navigate the delicate vascular networks of the corpus cavernosum, delivering stem cells to treat erectile dysfunction with a precision that standard injections cannot match [132]. Looking forward, the field is moving toward reconfigurable soft machines that can adapt to the unstructured terrain of the urinary tract. Recent innovations in ferrofluidic wetting [140] and modular solid-droplet systems [141] demonstrate how soft robots can split, fuse, and navigate narrow channels. Additionally, bioinspired colloidal collectives [142] and bimodal droplets [143] capable of 3D drifting offer new paradigms for maneuvering swarms in fluid-filled cavities like the bladder.

As these technologies mature, the integration of 4D-printed smart materials, such as stents that self-expand or degrade on command, will further blur the line between device and robot [144]. Ultimately, successful translation will depend on developing systems that do not just perform novel physics, but solve high-burden clinical problems with superior efficacy and safety compared to the current standard of care.

## 4. Conclusions

The application of Active Propelled Micro Technologies in urology represents a frontier with transformative potential. The urinary system, with its accessible fluid-filled cavities, specific chemical environment (urea), and amenability to standard imaging, is arguably the most “robot-friendly” system in the human body.

The transition from passive instillation to active robotic delivery addresses the fundamental limitations of current urologic therapies: washout in bladder cancer, invasiveness in stone management, and biofilm resistance in infections. Technologies such as urease-powered nanomotors for stone dissolution and burrowing immunobots for bladder cancer have shown convincing proof-of-concept efficacy in vivo.

However, the path to clinical adoption requires a concerted shift from “performance-based” research (focusing on speed and force) to “solution-based” translational engineering. This entails prioritizing biodegradability, ensuring compatibility with standard urologic imaging (fluoroscopy/ultrasound), and navigating the complex regulatory landscape of combination products. As fabrication methods like 2PP scale up and imaging modalities like MPI mature, active microrobots are poised to evolve from laboratory curiosities into essential tools in the urologist’s armamentarium, offering a future of precision, incision-free intervention for millions of patients worldwide.

## Figures and Tables

**Table 1:** Comparative Analysis of Propulsion Mechanisms for Urologic Applications.

Primary Classification	Sub-Classification/Mechanism	Operating Principle & Examples	Key Advantages & Applications	Limitations/Challenges
**Chemical Propulsion** (Autonomous “Engines”)	**Self-Electrophoresis**	**Principle:** Bimetallic nanorods (e.g., Pt-Au) in H_2_O_2_ create local electric fields via redox reactions.	Pioneering synthetic nanomotor mechanism.	**High Sensitivity to Ionic Strength:** Fails in urine/blood due to electric double layer collapse.
	**Self-Diffusiophoresis**	**Principle:** Asymmetric solute concentration gradient creates pressure imbalance/osmotic flow. **Ex:** Silica-based motors; Enzyme-coated particles.	**Salt Tolerance:** Robust in physiological fluids (urine, serum) unlike electrophoresis.	Requires specific chemical fuels.
	**Bubble Propulsion**	**Principle:** Rapid gas ejection (Jet propulsion). **Fuels:** H_2_O_2_ (O_2_ bubbles); Acid + Zn/Mg H_2_ bubbles).	**High Power:** High velocity; can tow cellular cargo. **App:** Useful in bladder (ultrasound contrast).	**Embolism Risk:** Dangerous in the bloodstream; safer in urinary tract.
	**Enzyme-Driven**	**Principle:** Biocompatible enzymes catalyze physiological fuels. **Ex:** Urease (Urea), Glucose Oxidase (Glucose), Catalase (Peroxide).	**Biocompatibility:** Biodegradable; uses endogenous fuels. **App:** Urease motors ideal for urology.	Dependent on local substrate concentration.
**External Field Propulsion** (Fuel-Free)	**Magnetic Propulsion**	**Types:** 1. **Helical:** Corkscrew motion via Rotating Magnetic Field (RMF). 2. **Surface Walkers:** Tumble/walk on tissue. 3. **Gradient Pulling:** Direct magnetic pull.	**Deep Penetration:** Tissue is transparent to magnetic fields. **Control:** Precise steering. **App:** Penetrating mucus/clots (helical).	Gradient pulling is weak for small scales; RMF preferred.
	**Acoustic Propulsion**	**Principle:** Ultrasound waves cause acoustic streaming or radiation force on asymmetric shapes (e.g., metallic nanowires).	**Clinical Compatibility:** Fits existing urology workflows. **Deep Penetration:** Reaches kidneys. **Safety:** Stable cavitation ensures tissue safety.	Requires coupling medium.
	**Light Propulsion**	**Principle:** Photonic energy via thermophoresis (heat) or photocatalysis (TiO_2_).	**Precision:** Localized control. **App:** Superficial lesions or endoscopic intra-organ use.	**Low Penetration:** Limited by tissue depth (even in NIR window).
**Biohybrid Propulsion** (Synthetic + Biological)	**Microorganism-Based**	**Bacteria:** (e.g., *S. typhimurium*, *E. coli*) Chemotaxis toward tumors. **Fungi:** (e.g., Yeast/JYC-robots) Coated with Fe_3_O_4_/ZIF-67.	**Tumor Homing:** Bacteria target hypoxic cores. **Detoxification:** Yeast absorbs mycotoxins. **App:** Oncology and environmental detox.	Biofouling risk in blood (lesser in urine).
	**Mammalian Cell-Based**	**Sperm:** Flagellar propulsion; mucus penetration. **Immune Cells:** (Macrophages/Neutrophils) Chemotaxis to inflammation; cross barriers. **293T/Tumor Cells:** Virus carriers; surface rolling.	**Barriers:** Crossing biological barriers (diapedesis). **Synergy:** Immune regulation + drug delivery.	Complex fabrication and maintenance of cell viability.
	**Herb Spore-Based**	**Principle:** Plant spores/pollen (e.g., *Spora Lygodii*, *Pollen Typhae*) as natural porous capsules.	**Stability:** Non-living (easy storage/transport). **Structure:** Natural porosity and mucoadhesion. **App:** Hemostasis, litholytic properties.	Lack active biological motility (rely on magnetic coating).

## Data Availability

No new data were created or analyzed in this study.

## References

[1] Sisodiya S.M. (2021). Precision medicine and therapies of the future. Epilepsia.

[2] Wang R.C., Wang Z. (2023). Precision medicine: Disease subtyping and tailored treatment. Cancers.

[3] Lou J., Duan H., Qin Q., Teng Z., Gan F., Zhou X., Zhou X. (2023). Advances in oral drug delivery systems: Challenges and opportunities. Pharmaceutics.

[4] Iacovacci V., Diller E., Ahmed D., Menciassi A. (2024). Medical microrobots. Annu. Rev. Biomed. Eng..

[5] Jiang J., Yang Z., Ferreira A., Zhang L. (2022). Control and autonomy of microrobots: Recent progress and perspective. Adv. Intell. Syst..

[6] Ezike T.C., Okpala U.S., Onoja U.L., Nwike C.P., Ezeako E.C., Okpara O.J., Okoroafor C.C., Eze S.C., Kalu O.L., Odoh E.C. (2023). Advances in drug delivery systems, challenges and future directions. Heliyon.

[7] Pal S., Naveen D., Tejpal, Debroy S. (2025). Introduction to Drug Delivery System: Past, Present, and Future Perspectives. Next-Generation Drug Delivery Systems.

[8] Stone M.B., Yaseen Z.S., Miller B.J., Richardville K., Kalaria S.N., Kirsch I. (2022). Response to acute monotherapy for major depressive disorder in randomized, placebo controlled trials submitted to the US Food and Drug Administration: Individual participant data analysis. BMJ.

[9] Han H.S., Koo S.Y., Choi K.Y. (2022). Emerging nanoformulation strategies for phytocompounds and applications from drug delivery to phototherapy to imaging. Bioact. Mater..

[10] Pan J., Wang Y., Chen Y., Zhang C., Deng H., Lu J., Chen W. (2025). Emerging strategies against accelerated blood clearance phenomenon of nanocarrier drug delivery systems. J. Nanobiotechnol..

[11] Prakash A., Malviya R., Sridhar S.B. (2025). Targeting the Reticuloendothelial System for the Management of Neurological Disorders. Curr. Drug Ther..

[12] Plaunt A.J., Nguyen T.L., Corboz M.R., Malinin V.S., Cipolla D.C. (2022). Strategies to overcome biological barriers associated with pulmonary drug delivery. Pharmaceutics.

[13] Hu M., Li X., You Z., Cai R., Chen C. (2024). Physiological barriers and strategies of lipid-based nanoparticles for nucleic acid drug delivery. Adv. Mater..

[14] Feng Y., An M., Liu Y., Sarwar M.T., Yang H. (2023). Advances in chemically powered micro/nanorobots for biological applications: A review. Adv. Funct. Mater..

[15] Hogg T. (2021). Acoustic power management by swarms of microscopic robots. J. Micro-Bio Robot..

[16] Hou Y., Wang H., Fu R., Wang X., Yu J., Zhang S., Huang Q., Sun Y., Fukuda T. (2023). A review on microrobots driven by optical and magnetic fields. Lab Chip.

[17] Preetam S., Pritam P., Mishra R., Rustagi S., Lata S., Malik S. (2024). Empowering tomorrow’s medicine: Energy-driven micro/nano-robots redefining biomedical applications. Mol. Syst. Des. Eng..

[18] Tian M., Keshavarz M., Demircali A.A., Han B., Yang G.-Z. (2025). Localized Microrobotic Delivery of Enzyme-Responsive Hydrogel-Immobilized Therapeutics to Suppress Triple-Negative Breast Cancer. Small.

[19] Ishikawa T. (2024). Fluid dynamics of squirmers and ciliated microorganisms. Annu. Rev. Fluid Mech..

[20] Jiang H., Costello J.H., Colin S.P. (2021). Fluid dynamics and efficiency of colonial swimming via multijet propulsion at intermediate Reynolds numbers. Phys. Rev. Fluids.

[21] Wu C., Omori T., Ishikawa T. (2024). Drag force on a microrobot propelled through blood. Commun. Phys..

[22] Folio D., Ferreira A. (2022). Modeling and estimation of self-phoretic magnetic Janus microrobot with uncontrollable inputs. IEEE Trans. Control Syst. Technol..

[23] Soto F., Karshalev E., Zhang F., Esteban Fernandez de Avila B., Nourhani A., Wang J. (2021). Smart materials for microrobots. Chem. Rev..

[24] Nelson B.J., Pané S. (2023). Delivering drugs with microrobots. Science.

[25] Saldana M., Gallegos S., Gálvez E., Castillo J., Salinas-Rodríguez E., Cerecedo-Sáenz E., Hernández-Ávila J., Navarra A., Toro N. (2024). The Reynolds Number: A Journey from Its Origin to Modern Applications. Fluids.

[26] Schumacher J., Sreenivasan K.R., Yakhot V. (2007). Asymptotic exponents from low-Reynolds-number flows. New J. Phys..

[27] Bagchi P., Balachandar S. (2003). Inertial and viscous forces on a rigid sphere in straining flows at moderate Reynolds numbers. J. Fluid Mech..

[28] Lauga E. (2007). Continuous breakdown of Purcell’s scallop theorem with inertia. Phys. Fluids.

[29] Jia L., Su G., Zhang M., Wen Q., Wang L., Li J. (2025). Propulsion Mechanisms in Magnetic Microrobotics: From Single Microrobots to Swarms. Micromachines.

[30] Ju X., Chen C., Oral C.M., Sevim S., Golestanian R., Sun M., Bouzari N., Lin X., Urso M., Nam J.S. (2025). Technology Roadmap of Micro/Nanorobots. ACS Nano.

[31] Somasundar A., Sen A. (2021). Chemically propelled nano and micromotors in the body: Quo vadis?. Small.

[32] Sánchez S., Soler L., Katuri J. (2015). Chemically powered micro-and nanomotors. Angew. Chem. Int. Ed..

[33] Zhang Z., Li Q., Cao W., Wei J., He L., Zheng X., Li X. (2025). Self-electrophoresis-propelled and self-built electric field-enhanced photocatalytic nanomotors for round-the-clock environmental remediation. Mater. Horiz..

[34] Pu R., Yang X., Mu H., Xu Z., He J. (2024). Current status and future application of electrically controlled micro/nanorobots in biomedicine. Front. Bioeng. Biotechnol..

[35] Moo J.G.S., Mayorga-Martinez C.C., Wang H., Khezri B., Teo W.Z., Pumera M. (2017). Nano/microrobots meet electrochemistry. Adv. Funct. Mater..

[36] Chen K., Chen T., Liu H., Yang Z. A Pt/Au hybrid self-actuating nanorobot towards to durg delivery system. Proceedings of the 10th IEEE International Conference on Nano/Micro Engineered and Molecular Systems.

[37] Zheng L., Hart N., Zeng Y. (2023). Micro-/nanoscale robotics for chemical and biological sensing. Lab Chip.

[38] Benavente F., van der Heijden R., Tjaden U.R., van der Greef J., Hankemeier T. (2006). Metabolite profiling of human urine by CE-ESI-MS using separation electrolytes at low pH. Electrophoresis.

[39] Yadav V. (2015). Self-Propelled Systems for Versatile Applications. Ph.D. Thesis.

[40] Asmolov E.S., Nizkaya T.V., Vinogradova O.I. (2022). Self-diffusiophoresis of Janus particles that release ions. Phys. Fluids.

[41] Yang F., Qian S., Zhao Y., Qiao R. (2016). Self-diffusiophoresis of Janus catalytic micromotors in confined geometries. Langmuir.

[42] Yan M., Xie L., Tang J., Liang K., Mei Y., Kong B. (2021). Recent advances in heterosilica-based micro/nanomotors: Designs, biomedical applications, and future perspectives. Chem. Mater..

[43] Shim S. (2022). Diffusiophoresis, diffusioosmosis, and microfluidics: Surface-flow-driven phenomena in the presence of flow. Chem. Rev..

[44] Zhou Y., Dai L., Jiao N. (2022). Review of bubble applications in microrobotics: Propulsion, manipulation, and assembly. Micromachines.

[45] Lee J.G., Raj R.R., Thome C.P., Day N.B., Martinez P., Bottenus N., Gupta A., Wyatt Shields C. (2023). Bubble-Based Microrobots With Rapid Circular Motions for Epithelial Pinning and Drug Delivery. Small.

[46] Das S., Steager E.B., Stebe K.J., Kumar V. Simultaneous control of spherical microrobots using catalytic and magnetic actuation. Proceedings of the 2017 International Conference on Manipulation, Automation and Robotics at Small Scales (MARSS).

[47] Ussia M., Urso M., Kratochvilova M., Navratil J., Balvan J., Mayorga-Martinez C.C., Vyskocil J., Masarik M., Pumera M. (2023). Magnetically driven self-degrading zinc-containing cystine microrobots for treatment of prostate cancer. Small.

[48] Zhang G., Yang S., Yang J.F., Gonzalez-Medrano D., Miskin M.Z., Koman V.B., Zeng Y., Li S.X., Kuehne M., Liu A.T. (2024). High energy density picoliter-scale zinc-air microbatteries for colloidal robotics. Sci. Robot..

[49] Chen C., Karshalev E., Guan J., Wang J. (2018). Magnesium-based micromotors: Water-powered propulsion, multifunctionality, and biomedical and environmental applications. Small.

[50] Xu C., Wang S., Wang H., Liu K., Zhang S., Chen B., Liu H., Tong F., Peng F., Tu Y. (2021). Magnesium-based micromotors as hydrogen generators for precise rheumatoid arthritis therapy. Nano Lett..

[51] Feng Y., Chang X., Liu H., Hu Y., Li T., Li L. (2021). Multi-response biocompatible Janus micromotor for ultrasonic imaging contrast enhancement. Appl. Mater. Today.

[52] Liu X., Wang Y., Peng Y., Shi J., Chen W., Wang W., Ma X. (2023). Urease-powered micromotors with spatially selective distribution of enzymes for capturing and sensing exosomes. ACS Nano.

[53] Tang S., Zhang F., Gong H., Wei F., Zhuang J., Karshalev E., Esteban-Fernández de Ávila B., Huang C., Zhou Z., Li Z. (2020). Enzyme-powered Janus platelet cell robots for active and targeted drug delivery. Sci. Robot..

[54] Zhou H., Liu Q., Chen M., Xie Y., Xu W., Zhang X., Jiang C., Dou P., Fang Z., Wang H. (2025). Urease-Driven Janus Nanomotors for Dynamic Enrichment and Multiplexed Detection of Bladder Cancer MicroRNAs in Urine. ACS Sens..

[55] Amiri Z., Hasani A., Abedini F., Malek M., Madaah Hosseini H.R. (2024). Urease-powered black TiO_2_ micromotors for photothermal therapy of bladder cancer. ACS Appl. Mater. Interfaces.

[56] Qu Q., Cheng W., Zhang X., Ravanbakhsh H., Tang G., Zhou A., Pei D., Xiong R., Huang C. (2023). Glucose-responsive enzymatic cascade microreactors in gas-shearing microfluidics microcapsules. Adv. Mater. Technol..

[57] Yang S., He Q., Lin X. (2025). Dual chemotaxis of glucose oxidase-powered nanomotors towards the concentration gradients of both glucose and proton. Colloids Surf. A Physicochem. Eng. Asp..

[58] Serra-Casablancas M., Di Carlo V., Esporrin-Ubieto D., Prado-Morales C., Bakenecker A.C., Sanchez S. (2024). Catalase-powered nanobots for overcoming the mucus barrier. ACS Nano.

[59] Wang Q., Zhang L. (2021). External power-driven microrobotic swarm: From fundamental understanding to imaging-guided delivery. ACS Nano.

[60] Xu T., Gao W., Xu L.P., Zhang X., Wang S. (2017). Fuel-free synthetic micro-/nanomachines. Adv. Mater..

[61] Zhu S., Chen Y., Liu G., Qian H., Niu F., Wang Y., Zhao Y., Luo T., Yang R. (2022). External Field-Driven Untethered Microrobots for Targeted Cargo Delivery. Adv. Mater. Technol..

[62] Yang Q., Tang S., Lu D., Li Y., Wan F., Li J., Chen Q., Cong Z., Zhang X., Wu S. (2022). Pollen Typhae-Based Magnetic-Powered Microrobots toward Acute Gastric Bleeding Treatment. ACS Appl. Bio Mater..

[63] Peyer K.E., Zhang L., Nelson B.J. (2013). Bio-inspired magnetic swimming microrobots for biomedical applications. Nanoscale.

[64] Hou Y., Bai K., Zhong S., Zheng Z., Shi Q., Huang Q., Fukuda T., Li F., Wang H. (2025). Swimming performance enhancement of the magnetic helical microrobots based on surface microstructure modification. IEEE Robot. Autom. Lett..

[65] Li T., Zhang A., Shao G., Wei M., Guo B., Zhang G., Li L., Wang W. (2018). Janus microdimer surface walkers propelled by oscillating magnetic fields. Adv. Funct. Mater..

[66] Jia Y., Liao P., Wang Y., Sun D. (2022). Magnet-Driven Microwalker in Surface Motion Based on Frictional Anisotropy. Adv. Intell. Syst..

[67] Diller E., Giltinan J., Sitti M. (2013). Independent control of multiple magnetic microrobots in three dimensions. Int. J. Robot. Res..

[68] Ryan P., Diller E. Five-degree-of-freedom magnetic control of micro-robots using rotating permanent magnets. Proceedings of the 2016 IEEE International Conference on Robotics and Automation (ICRA).

[69] Xiao Y., Zhang J., Fang B., Zhao X., Hao N. (2022). Acoustics-actuated microrobots. Micromachines.

[70] Valdez-Garduño M., Leal-Estrada M., Oliveros-Mata E.S., Sandoval-Bojorquez D.I., Soto F., Wang J., Garcia-Gradilla V. (2020). Density asymmetry driven propulsion of ultrasound-powered Janus micromotors. Adv. Funct. Mater..

[71] Deng Y., Paskert A., Zhang Z., Wittkowski R., Ahmed D. (2023). An acoustically controlled helical microrobot. Sci. Adv..

[72] Salib A., Halpern E., Eisenbrey J., Chandrasekar T., Chung P.H., Forsberg F., Trabulsi E.J. (2023). The evolving role of contrast-enhanced ultrasound in urology: A review. World J. Urol..

[73] Cranston D., Leslie T., Ter Haar G. (2021). A Review of High-Intensity Focused Ultrasound in Urology. Cancers.

[74] Bunea A.I., Martella D., Nocentini S., Parmeggiani C., Taboryski R., Wiersma D.S. (2021). Light-Powered Microrobots: Challenges and Opportunities for Hard and Soft Responsive Microswimmers. Adv. Intell. Syst..

[75] Yang W.G., Wang X.W., Wang Z., Liang W.F., Ge Z.X. (2023). Light-powered microrobots: Recent progress and future challenges. Opt. Laser. Eng..

[76] Ullattil S.G., Pumera M. (2023). Light-Powered Self-Adaptive Mesostructured Microrobots for Simultaneous Microplastics Trapping and Fragmentation via in situ Surface Morphing. Small.

[77] Zarepour A., Khosravi A., Iravani S., Zarrabi A. (2024). Biohybrid Micro/Nanorobots: Pioneering the Next Generation of Medical Technology. Adv. Healthc. Mater..

[78] Li Y., Tang S., Cong Z., Lu D., Yang Q., Chen Q., Zhang X., Wu S. (2022). Biohybrid bacterial microswimmers with metal-organic framework exoskeletons enable cytoprotection and active drug delivery in a harsh environment. Mater. Today Chem..

[79] Cao Z., Liu J. (2024). Coated bacteria: Advanced living materials for microbial therapy. Acc. Mater. Res..

[80] Cong Z., Tang S., Xie L., Yang M., Li Y., Lu D., Li J., Yang Q., Chen Q., Zhang Z. (2022). Magnetic-Powered Janus Cell Robots Loaded with Oncolytic Adenovirus for Active and Targeted Virotherapy of Bladder Cancer. Adv. Mater..

[81] Yan X., Zhou Q., Yu J., Xu T., Deng Y., Tang T., Feng Q., Bian L., Zhang Y., Ferreira A. (2015). Magnetite nanostructured porous hollow helical microswimmers for targeted delivery. Adv. Funct. Mater..

[82] Yan X., Zhou Q., Vincent M., Deng Y., Yu J., Xu J., Xu T., Tang T., Bian L., Wang Y.-X.J. (2017). Multifunctional biohybrid magnetite microrobots for imaging-guided therapy. Sci. Robot..

[83] Chen Q., Tang S., Li Y., Cong Z., Lu D., Yang Q., Zhang X., Wu S. (2021). Multifunctional metal–organic framework exoskeletons protect biohybrid sperm microrobots for active drug delivery from the surrounding threats. ACS Appl. Mater. Interfaces.

[84] Cong Z., Li Y., Xie L., Chen Q., Tang M., Thongpon P., Jiao Y., Wu S. (2024). Engineered Microrobots for Targeted Delivery of Bacterial Outer Membrane Vesicles (OMV) in Thrombus Therapy. Small.

[85] Li Y., Cong Z., Xie L., Tang S., Ren C., Peng X., Tang D., Wan F., Han H., Zhang X. (2023). Magnetically Powered Immunogenic Macrophage Microrobots for Targeted Multimodal Cancer Therapy. Small.

[86] Xie L., Cong Z., Tang S., Yang M., Li Y., Ren C., Chen Q., Lu D., Wan F., Zhang X. (2023). Oncolytic adenovirus-loaded magnetic-driven Janus tumor cell robots for active and targeted virotherapy of homologous carcinoma. Mater. Today Chem..

[87] Yang Q., Yuan W., Zhao T., Jiao Y., Tang M., Cong Z., Wu S. (2024). Magnetic-Powered Spora Lygodii Microrobots Loaded with Doxorubicin for Active and Targeted Therapy of Bladder Cancer. Drug Des. Dev. Ther..

[88] Kumar S., Pollok R., Goldsmith D. (2023). Renal and urological disorders associated with inflammatory bowel disease. Inflamm. Bowel Dis..

[89] Brouwer O.R., Albersen M., Parnham A., Protzel C., Pettaway C.A., Ayres B., Antunes-Lopes T., Barreto L., Campi R., Crook J. (2023). European Association of Urology-American Society of Clinical Oncology collaborative guideline on penile cancer: 2023 update. Eur. Urol..

[90] Knoll N., Gralla O. (2024). Urologic Diseases. Encyclopedia of Quality of Life and Well-Being Research.

[91] Lorusso G., Assumma S., Gavi F., Panio E., Turri F., Fettucciari D., Sanesi D., Schubert O., Bracco M., Russo P. (2025). Urology in the digital age: The power of telemedicine. Urol. J..

[92] Han S., Zhao S., Zhong R., Liu H., Liu L., Yan Y. (2025). An analysis of the burden of urolithiasis: Differences between the global, China, India and the United States, with projections through 2050. Urolithiasis.

[93] Awedew A.F., Han H., Berice B.N., Dodge M., Schneider R.D., Abbasi-Kangevari M., Al-Aly Z., Almidani O., Alvand S., Arabloo J. (2024). The global, regional, and national burden of urolithiasis in 204 countries and territories, 2000–2021: A systematic analysis for the Global Burden of Disease Study 2021. EClinicalMedicine.

[94] Papatsoris A., Geavlete B., Radavoi G.D., Alameedee M., Almusafer M., Ather M.H., Budia A., Cumpanas A.A., Kiremi M.C., Dellis A. (2025). Management of urinary stones by experts in stone disease (ESD 2025). Arch. Ital. Urol. Androl./Arch. Ital. Di Urol. Androl..

[95] Coughlan L.-A. (2024). Advancing Kidney Stone Management: A Technical and Commercial Review of Intraoperative Medical Device. Master’s Thesis.

[96] Wéber A., Vignat J., Shah R., Morgan E., Laversanne M., Nagy P., Kenessey I., Znaor A. (2024). Global burden of bladder cancer mortality in 2020 and 2040 according to GLOBOCAN estimates. World J. Urol..

[97] Gao K., Zhang S., Liu J., Zhang F., Liu N., Dong J., Zhang T., Gao J., Qin S., An J. (2025). Supportive care needs of the family caregivers of urostomy patients: A qualitative study. Sci. Rep..

[98] Yeary K.H.K., Yu H., Kuliszewski M.G., Li Q., McCann S.E., Pratt R., Saad-Harfouche F.G., Wang Z., Clark N., Wang C. (2024). Outcomes of a Dietary Intervention to Reduce Bladder Cancer Recurrence and Progression in Survivors of Non–Muscle-Invasive Bladder Cancer. J. Natl. Compr. Cancer Netw..

[99] Abbas S., Soomro N., Shafik R., Heer R., Adhikari K. (2025). Attention-enabled Explainable AI for Bladder Cancer Recurrence Prediction. arXiv.

[100] Kumbham S., Md Mahabubur Rahman K., Foster B.A., You Y. (2025). A comprehensive review of current approaches in bladder cancer treatment. ACS Pharmacol. Transl. Sci..

[101] Wu Y., Gu X., Chen X., Cui Y., Jiang W., Liu B. (2024). Hydrogel: A new material for intravesical drug delivery after bladder cancer surgery. J. Mater. Chem. B.

[102] Hu W., Zhou Z., Zou F., Huang Y., Li M., Zhang Y., Sun K., Deng H., Cheng F., Zhao Z. (2025). Harnessing Natural Pollen as Sustained-Release, Mucoadhesive, and Biosafe Drug Microcapsules for Intravesical Instillation in Bladder Cancer Treatment. Small.

[103] Liu Z. (2021). PPARγ: New Insights into Its Role in Urothelial Differentiation and Regeneration. Ph.D. Thesis.

[104] Kolawa A., D’Souza A., Tulpule V. (2023). Overview, diagnosis, and perioperative systemic therapy of upper tract urothelial carcinoma. Cancers.

[105] Bitaraf M., Ghafoori Yazdi M., Amini E. (2023). Upper tract urothelial carcinoma (UTUC) diagnosis and risk stratification: A comprehensive review. Cancers.

[106] Oh C., Jung H.N., Park J., Awad J.M., Jiang D., Dowling D.J., Im H.-J. (2025). Nanomedicine and Spleen-Targeting Strategies for Precision Immunomodulation: Advances, Challenges, and Future Perspectives. ACS Nano.

[107] Zheng S., Carugo D., Mosayyebi A., Turney B., Burkhard F., Lange D., Obrist D., Waters S., Clavica F. (2021). Fluid mechanical modeling of the upper urinary tract. WIREs Mech. Dis..

[108] Wang S., Jin S., Shu Q., Wu S. (2021). Strategies to get drugs across bladder penetrating barriers for improving bladder cancer therapy. Pharmaceutics.

[109] Marchenko I.V., Trushina D.B. (2023). Local drug delivery in bladder cancer: Advances of nano/micro/macro-scale drug delivery systems. Pharmaceutics.

[110] Liu D., Wang T., Lu Y. (2022). Untethered microrobots for active drug delivery: From rational design to clinical settings. Adv. Healthc. Mater..

[111] Wang J., Dong Y., Ma P., Wang Y., Zhang F., Cai B., Chen P., Liu B.F. (2022). Intelligent micro-/nanorobots for cancer theragnostic. Adv. Mater..

[112] Dekanovsky L., Ying Y., Zelenka J., Plutnar J., Beladi-Mousavi S.M., Křížová I., Novotný F., Ruml T., Pumera M. (2022). Fully programmable collective behavior of light-powered chemical microrobotics: pH-dependent motion behavior switch and controlled cancer cell destruction. Adv. Funct. Mater..

[113] Khabbazian A., Kwong L., Lewis A., Liu E., Abdelrazec N., Bakenecker A.C., Fontanals N., Lopez G., Sánchez S., Lopez J.M. (2025). Kidney Stone Dissolution By Tetherless, Enzyme-Loaded, Soft Magnetic Miniature Robots. Adv. Healthc. Mater..

[114] Habibnejad-Korayem M., Nabiei O., Gharibshah S., Nouhi-Hefzabad R. (2021). Atomistic assessment of cystine kidney stone behavior in a mechanical breakdown process by nanobiorobots through classical molecular dynamics simulations. J. Phys. Chem. B.

[115] Zhang Y., Zhang Y., Han Y., Gong X. (2022). Micro/nanorobots for medical diagnosis and disease treatment. Micromachines.

[116] Dogan N.O., Suadiye E., Wrede P., Lazovic J., Dayan C.B., Soon R.H., Aghakhani A., Richter G., Sitti M. (2024). Immune cell-based microrobots for remote magnetic actuation, antitumor activity, and medical imaging. Adv. Healthc. Mater..

[117] Choi H., Jeong S.H., Simo C., Bakenecker A., Liop J., Lee H.S., Kim T.Y., Kwak C., Koh G.Y., Sanchez S. (2024). Urease-powered nanomotor containing STING agonist for bladder cancer immunotherapy. Nat. Commun..

[118] Jia J., Wu G., Zhang H., Wang F., Gu X., Dorma D., Zhang L., Chen H., Xu Y., Xie H. (2025). Magnetic-Driven Hydrogel Robots Loaded with Mitomycin for Active Therapy of Bladder Cancer. ACS Appl. Mater. Interfaces.

[119] Sun J., Chu R., Wu X., Yu Q., Xiao W., Ao H., Wang Y., Wu T., Ju H., Wu J. (2025). Anti-biopassivated Reticular Micromotors for Bladder Cancer Therapy. J. Am. Chem. Soc..

[120] Dogan N.O., Ceylan H., Suadiye E., Sheehan D., Aydin A., Yasa I.C., Wild A.M., Richter G., Sitti M. (2022). Remotely Guided Immunobots Engaged in Anti-Tumorigenic Phenotypes for Targeted Cancer Immunotherapy. Small.

[121] Hsiao C.H., Lin Y.W., Liu C.H., Nguyen H.T., Chuang A.E. (2024). Light-Driven Green-Fabricated Artificial Intelligence-Enabled Micro/Nanorobots for Multimodal Phototherapeutic Management of Bladder Cancer. Adv. Heal. Mater..

[122] Han H., Ma X.T., Deng W.T., Zhang J.H., Tang S.S., Pak O.S., Zhu L.L., Criado-Hidalgo E., Gong C., Karshalev E. (2024). Imaging-guided bioresorbable acoustic hydrogel microrobots. Sci. Robot..

[123] Tang T., Kong S., Xie J., Deng Q., Lai C., Guo S., Yu H., Zhou J. (2025). Prolonged Release of Bacillus Calmette-Guerin by Floating Microbubbles to Enhance Intravesical Immunotherapy for Bladder Cancer. ACS Nano.

[124] Adejokun S.A., Kumat S.S., Shiakolas P.S. (2023). A Microrobot With an Attached Microforce Sensor for Transurethral Access to the Bladder Interior Wall. J. Eng. Sci. Med. Diagn. Ther..

[125] Choi H., Kim B., Seo Y., Kim T.Y., Bong K.W., Hahn S.K. (2026). Magnetically controlled microgelbots with stem cells for the treatment of interstitial cystitis. Biomaterials.

[126] Hu Q., Wu Z., Tian Y., Wang J., Pan Z., Yu Y., Cheng Y., Yang Y., Tang H., Zang J. (2025). A magnetic soft robotic system for intelligent bladder volume control. npj Flex. Electron..

[127] Peng X., Tang H., Zhao Z., Zheng Y., Gui X., Jiang A., He P., Wen X., Zhang Q., Mei Z. (2024). Intelligent Generic High-Throughput Oscillatory Shear Technology Fabricates Programmable Microrobots for Real-Time Visual Guidance During Embolization. Small.

[128] Peng Q., Wang S., Han J., Huang C., Yu H., Li D., Qiu M., Cheng S., Wu C., Cai M. (2024). Thermal and Magnetic Dual-Responsive Catheter-Assisted Shape Memory Microrobots for Multistage Vascular Embolization. Research.

[129] Peng X., Urso M., Balvan J., Masarik M., Pumera M. (2022). Self-Propelled Magnetic Dendrite-Shaped Microrobots for Photodynamic Prostate Cancer Therapy. Angew. Chem. Int. Ed. Engl..

[130] Villa K., Sopha H., Zelenka J., Motola M., Dekanovsky L., Beketova D.C., Macak J.M., Ruml T., Pumera M. (2022). Enzyme-Photocatalyst Tandem Microrobot Powered by Urea for Escherichia coli Biofilm Eradication. Small.

[131] Mostaghaci B., Yasa O., Zhuang J., Sitti M. (2017). Bioadhesive Bacterial Microswimmers for Targeted Drug Delivery in the Urinary and Gastrointestinal Tracts. Adv. Sci..

[132] Wang S., Wang Z., Shen Z., Zhang M., Jin D., Zheng K., Liu X., Chai M., Wang Z., Chi A. (2024). Magnetic soft microrobots for erectile dysfunction therapy. Proc. Natl. Acad. Sci. USA.

[133] Dillinger C., Rasaiah A., Vogel A., Bahou C., Monastyrskaya K., Gheinani A.H., Ahmed D. (2025). Real-time color flow mapping of ultrasound microrobots. Sci. Adv..

[134] Luk E., Wong B., Serracin L.G., Trieu A., Tondat A.M., Laing A., Magdanz V. RENAL: Robot Enhanced Navigation and Localization. Proceedings of the 2025 International Conference on Manipulation, Automation and Robotics at Small Scales (MARSS).

[135] Ceylan H., Sinibaldi E., Misra S., Pasricha P.J., Hutmacher D.W. (2025). Translating Milli/Microrobots with a Value-Centered Readiness Framework. arXiv.

[136] Nosrati H., Salehiabar M., Fridoni M., Abdollahifar M.-A., Kheiri Manjili H., Davaran S., Danafar H. (2019). New insight about biocompatibility and biodegradability of iron oxide magnetic nanoparticles: Stereological and in vivo MRI monitor. Sci. Rep..

[137] Pichler R., Diem G., Hackl H., Koutník J., Mertens L.S., DAndrea D., Pradere B., Soria F., Mari A., Laukhtina E. (2023). Intravesical BCG in bladder cancer induces innate immune responses against SARS-CoV-2. Front. Immunol..

[138] Xu T., Yu J., Yan X., Choi H., Zhang L. (2015). Magnetic actuation based motion control for microrobots: An overview. Micromachines.

[139] Alabay H.H., Le T.-A., Ceylan H. (2024). X-ray fluoroscopy guided localization and steering of miniature robots using virtual reality enhancement. Front. Robot. AI.

[140] Sun M., Hao B., Yang S., Wang X., Majidi C., Zhang L. (2022). Exploiting ferrofluidic wetting for miniature soft machines. Nat. Commun..

[141] Sun M., Wu Y., Zhang J., Zhang H., Liu Z., Li M., Wang C., Sitti M. (2024). Versatile, modular, and customizable magnetic solid-droplet systems. Proc. Natl. Acad. Sci. USA.

[142] Sun M., Yang S., Jiang J., Jiang S., Sitti M., Zhang L. (2023). Bioinspired self-assembled colloidal collectives drifting in three dimensions underwater. Sci. Adv..

[143] Sun M., Sun B., Park M., Yang S., Wu Y., Zhang M., Kang W., Yoon J., Zhang L., Sitti M. (2024). Individual and collective manipulation of multifunctional bimodal droplets in three dimensions. Sci. Adv..

[144] Ceylan H., Yasa I.C., Yasa O., Tabak A.F., Giltinan J., Sitti M. (2019). 3D-printed biodegradable microswimmer for theranostic cargo delivery and release. ACS Nano.

